# The application of artificial intelligence in health policy: a scoping review

**DOI:** 10.1186/s12913-023-10462-2

**Published:** 2023-12-15

**Authors:** Maryam Ramezani, Amirhossein Takian, Ahad Bakhtiari, Hamid R. Rabiee, Sadegh Ghazanfari, Hakimeh Mostafavi

**Affiliations:** 1 Department of Health Management, Policy and Economics, School of Public Health, Tehran University of Medical Sciences, Tehran, Iran; 2 Department of Global Health and Public Policy, School of Public Health, Tehran University of Medical Sciences, Tehran, Iran; 3 Health Equity Research Center (HERC), Tehran University of Medical Sciences, Tehran, Iran; 4 Department of Computer Engineering, Sharif University of Technology, Tehran, Iran

**Keywords:** Artificial intelligence, Health system, Policymaking, Health policy

## Abstract

**Background:**

Policymakers require precise and in-time information to make informed decisions in complex environments such as health systems. Artificial intelligence (AI) is a novel approach that makes collecting and analyzing data in complex systems more accessible. This study highlights recent research on AI’s application and capabilities in health policymaking.

**Methods:**

We searched PubMed, Scopus, and the Web of Science databases to find relevant studies from 2000 to 2023, using the keywords “artificial intelligence” and “policymaking.” We used Walt and Gilson’s policy triangle framework for charting the data.

**Results:**

The results revealed that using AI in health policy paved the way for novel analyses and innovative solutions for intelligent decision-making and data collection, potentially enhancing policymaking capacities, particularly in the evaluation phase. It can also be employed to create innovative agendas with fewer political constraints and greater rationality, resulting in evidence-based policies. By creating new platforms and toolkits, AI also offers the chance to make judgments based on solid facts. The majority of the proposed AI solutions for health policy aim to improve decision-making rather than replace experts.

**Conclusion:**

Numerous approaches exist for AI to influence the health policymaking process. Health systems can benefit from AI’s potential to foster the meaningful use of evidence-based policymaking.

**Supplementary Information:**

The online version contains supplementary material available at 10.1186/s12913-023-10462-2.

## Background

Worldwide, in a context characterized by fiscal conservatism, health systems face several intertwined challenges, such as the rising burden of non-communicable diseases (NCDs), population aging, increased demand for healthcare services, low productivity, and soaring healthcare expenditures [1]. These obstacles prevent health systems from achieving their fundamental goals, namely financial security and equitable access to healthcare services. Hence, an urgent need to transform healthcare systems to deal with such constraints. Conventionally, the research and policymaking communities have been following digital solutions, i.e., those for screening, diagnostic, and treatment services, which are far from universally implemented, particularly in low and middle-income countries (LMICs) [2]. On the other hand, the administration of digital solutions in other sectors has offered several opportunities for health systems to adopt novel approaches in policymaking, service provision, monitoring, forecasting, and simulating complex systems across various applications [3, 4]. This transition has been scaled up by increasing data availability and concurrent advances in IT infrastructures and mobile computing power, which boosted the implementation of some aspects of health systems’ digitalization during the COVID-19 pandemic [5].

Big data and artificial intelligence (AI) are widely used tools in several fields that allow planners to assess whether the intended policy/intervention will yield favorable outcomes. So far, the administration of AI has been focused more on delivering public services than policymaking [6], mainly due to the complexity and interdisciplinary nature of policymaking. As a result, digital solutions based on AI have been highly utilized for purposes such as sensing need patterns, developing evidence-based programs, and simulating and evaluating outcomes [7, 8]. The management of health conditions and their consequences at a public health policymaking level can benefit from different types of analysis of heterogeneous data, including the use of healthcare devices, physiological, cognitive, clinical, medication, personal, behavioral, lifestyle, and occupational, and environmental data [9].

Nevertheless, innovative digital solutions such as AI are a two-sided sword; and have the potential to improve governance and overall performance, while being able to violate citizens’ privacy and compromise fairness. In this regard, several research agendas have begun to evaluate the use of new approaches like big data, machine learning, blockchain, and AI in various fields. In particular, among various approaches, AI has become the core of digital transformation that led to a paradigm shift in many fields, including health policymaking. Public health is influenced by a variety of factors, including health systems, access to medicine, socioeconomic conditions, information systems and good governance. AI has a great potential to improve the capacity of these determinants [10–12]. The purpose of this research is to provide a comprehensive overview of AI applications in health policy. We anticipate that our findings will raise policymakers’ awareness of the potential of AI for better policymaking in the health systems.

## Methods

We conducted this review in line with the framework provided by Arksey and O’Malley [13]. In step one, we developed the research questions. The second step was to find relevant studies. We searched the PubMed, Scopus, and Web of Science databases for relevant studies published between 2000 and 2023 using the keywords “artificial intelligence” and “policy making.” In step 3, two researchers (MR and AB) independently screened the titles and abstracts of identified articles. In step 4, extracted data was charted, synthesized, and disagreements were resolved.

### Search strategy and inclusion criteria

The used keywords are listed in Table 1. We also mined Google Scholar to ensure the search was comprehensive. PubMed is primarily concerned with medical and health-related research. The other two databases assisted in identifying the studies in the field of engineering and social sciences, which we eventually combined from all three databases. Articles that discussed AI at the policymaking level and were published in English between 2000 and 2023 met our inclusion criteria.

**Table 1:** Databases and keywords

Databases	Query	Results
Web of Sciences	((((((((((TI=(policy)) OR TI=(“global health”)) OR TI=(“public health”)) OR TI=(“international health”)) OR TI=(policies)) OR TI=(“national reform”)) OR TI=(“priority setting”)) OR TI=(“national program”)) OR TI=(“public administration”)) OR TI=(“national strategy”)) (((((AB=(“deep learning”)) OR AB=(“artificial intelligence”)) OR AB=(“machine learning”)) OR AB=(“Big data”))OR AB=(“data mining”)) AB=(Health)	373
Scopus	((TITLE-ABS-KEY (“big data”) OR TITLE-ABS-KEY (“data mining”) OR TITLE-ABS-KEY (“deep learning”) OR TITLE-ABS-KEY (“artificial intelligence”) OR TITLE-ABS-KEY (“machine learning”))) AND (TITLE-ABS-KEY (health)) AND ((TITLE (policy) OR TITLE (“global health”) OR TITLE (“public health”) OR TITLE (“international health”) OR TITLE (policies) OR TITLE (“national reform”) OR TITLE (“priority setting”) OR TITLE (“national program”) OR TITLE (“public administration”) OR TITLE (“national strategy”)))	983
PubMed	(health[Title/Abstract]) AND ((((((((((((policy[Title]) OR (“global health” [Title])) OR (“public health” [Title])) OR (“international health” [Title])) OR (policies[Title])) OR (“national reform” [Title])) OR (“priority setting” [Title])) OR (“national program”[Title])) OR (“public administration”[Title])) OR (“national strategy”[Title]))) AND (((((“data mining”[Title/Abstract]) OR (“big data”[Title/Abstract])) OR (“artificial intelligence”[Title/Abstract])) OR (“deep learning”[Title/Abstract])) OR (“machine learning”[Title/Abstract])))	289

### Charting the data

Due to limited knowledge synthesis covering the topic, to develop an initial conceptual framework, we synthesized the data by placing similar codes into the categories of AI applications in health policy. Two authors (MR and AB) independently categorized AI applications and created descriptions by synthesizing the extracted information. We used the policy triangle framework by Walt and Gilson, i.e., content, process, actors, and context dimensions to chart the data, [14]. All authors reviewed and discussed the framework until we reached final consensus.

## Results

**Fig. 1:**
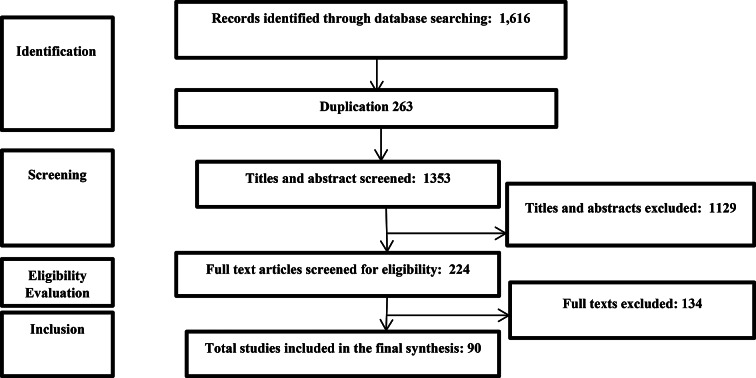
Search strategy results

We initially found 1616 articles, 263 of which were removed due to duplication. Based on the title and abstract analysis, we removed 1129 articles from the remaining 1353. After evaluating the full text of 224 articles, 90 articles were included in the final review (Please see Fig. 1 and Appendix 1 for details of included studies).

### The application of artificial intelligence in health policy

**Fig. 2:**
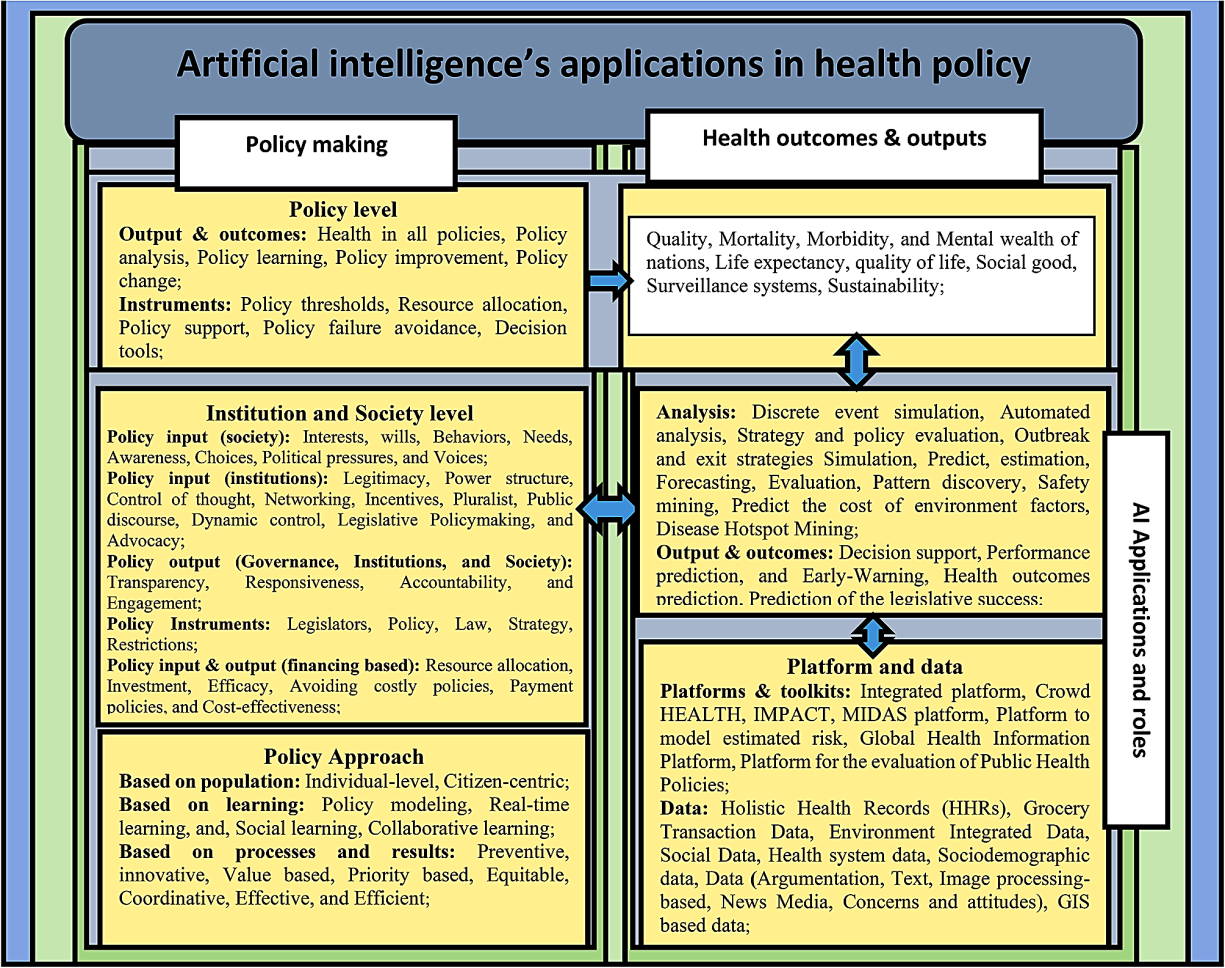
A Framework for AI-equipped policymaking: existing knowledge and future directions

As presented in Fig. 2, future studies can focus on applications and capabilities of AI to improve health policy by considering elements, i.e., approach, policy input, and policy output. Further, AI can follow health outcomes and outputs by changing these elements.

According to Fig. 2, applications and capabilities of AI include novel analysis, collecting new types of data, and paving the way for the Internet of Things (IoT), which can be used to address various problems.

As health policy is a complicated phenomenon with varied elements, we encourage future studies to consider the dimensions mentioned in Fig. 2, when providing policy options for real-time learning, addressing pandemics, or preventing NCDs. Therefore, recommendations for preventive policy options should be provided by future research to policymakers through the use of platforms that track key performance indicators (e.g., mortality, morbidity, and life expectancy). Meanwhile, researchers are recommended to consider factors such as interests, preferences, the voice of the society, accountability, and transparency of institutions when developing policies that intend to improve life expectancy, quality of life, and social good, by using AI applications and capabilities.

Interactions between the elements of the health policy triangle (content, process, actors, and context), equipped with AI applications and capabilities, has the capacity to change the policy process through new methods of data collection, analysis, and expert systems for decision-making. These instruments can help health system governance to make better and more informed decisions with novel approaches. Our findings illustrated the capability of AI to change the policy process, which might lead to changes in the context, content, and policy actors. By equipping health systems with such capabilities, policymakers can make decisions that facilitate achieving progressive goals.

**Fig. 3:**
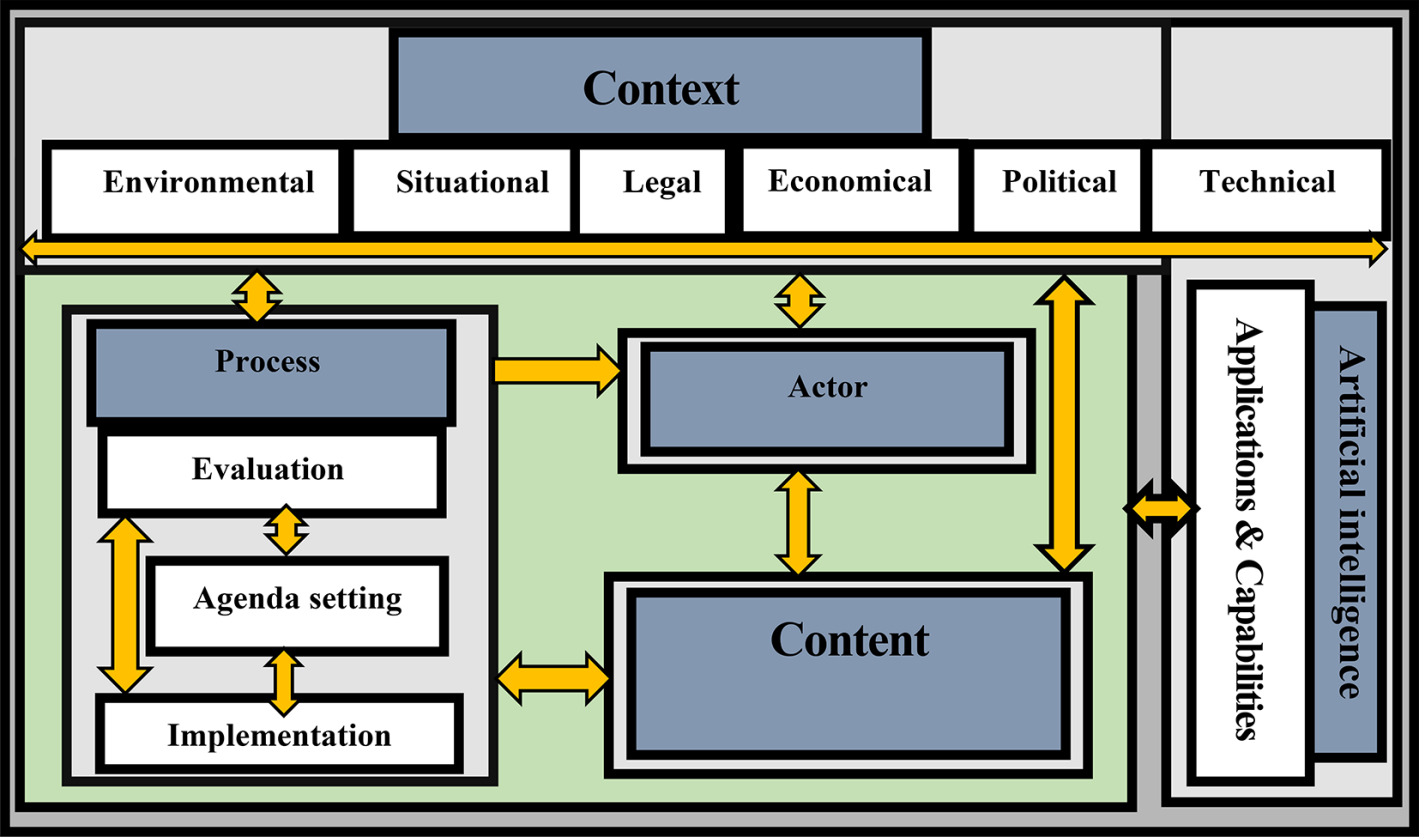
A Framework for Interactions between AI and the elements of health policy triangle

Figure 3 depicts potential interactions between AI and various dimensions of the health policy triangle, such as the ability to conduct a systematic analysis of the context, both internally and externally, i.e., the legal aspects of public policy that may affect the health system. CrowdHEALTH’s Health Policy Model, a new paradigm for health records, can include all current health data [15]. The model accomplishes this by integrating big data technologies throughout the entire data path, including mechanisms for causal and risk analysis [12], allowing for the development of policy models and the generation of analytical results for policymakers to make evidence-based decisions and evaluations toward a “health in all policies” approach [15–17]. Health Analytics Tools provide quantitative policy support and serve as a foundation for meta-analytic operations by the hierarchical structure of this model, which allows for versatility in the formulation and handling of the policies [15]. This comprehensive strategy organizes aggregate knowledge into clusters to provide a perception into various demographic groups based on various variables (e.g., location, occupation, medication status, emerging risks, etc.) [18]. The Policy Development Toolkit (PDT) is another tool that provides causal analysis by calculating total costs (expenses), forecasting information by evaluating the clinical effectiveness of reimbursement costs per medical condition, gender, and age for outpatient healthcare, and risk stratification by investigating screening parameters, indexes (Indicators), and other aspects of healthcare management [19]. As a result, PDT appears to be a promising option for policymakers who seek an effective decision-support system, which can improve HiAP (Health in All Policy Making) by assisting policymakers in aligning policies with their objectives, such as lowering healthcare costs, improving clinical efficacy, and preventing fraud [20]. Praedico is a customized surveillance and data analytics platform built on big data technologies that allow data exchange with other industries and sectors [21]. These platforms are linked to the fact that improved performance of health determinants translates into improved health. These platforms and toolkits serve as the foundation for such policies, which can result in more efficient healthcare, better health outcomes, patient-centered care, real-time learning and monitoring, reuse of existing data, and the provision of an actual source of data on the efficacy and affordability of medical interventions [20].

One study investigated the usability of the MIDAS (Meaningful Integration of Data Analytics and Services) Project, a big data platform for health policymaking with a number of international partners and pilot sites [22]. It supports health policy decision-making, public health activity planning, and the implementation of the HiAP approach [23]. Metamodeling is a crucial tool for decision-makers to examine policies with multivariate outcomes by making results from complex models accessible [24]. Another study that adopted a global viewpoint, introduced the Health Information Platform, which has two dimensions: to analyze and disseminate the theoretical and technological advancements of the primary research in global health and to monitor real-time emergencies, epidemic situations, and trending topics associated with global health. The platform aids in reducing the danger of infectious diseases occurring worldwide, developing effective health policies, and actively engaging in international global health issues [25].

Traditional methods such as pseudo-evaluation and formal evaluation are commonly used to facilitate the evaluation of public health policies. Similarly, another study proposed the use of a Big Data Platform to assist the formulation and evaluation of public health policies, allowing for evidence-based analysis and reducing the need for costly clinical trials [26]. A Big Data framework is appropriate for policymaking because it allows: (i) timely response, fostering interactions among policymakers, (ii) parallel mining of tasks, and (iii) supporting automatic monitoring and evaluation of a given policy [26]. An additional tool for modeling and simulating public health policy is IMPACT, which has demonstrated the capacity to connect policymakers and health policy-modellers within an integrated framework. This facilitates the sharing, upkeep, reuse, and deployment of population-based health models [27].

Some of these tools, platforms, and integrated systems have been developed to address certain risks or concerns, but they can also be upgraded to address additional risks and issues. For instance, EVOTION (developed to manage hearing impairments) is a new European research and innovation project that aim to develop an integrated platform that: (a) enables the identification of causal and other effects, (b) the selection of effective interventions based on the outcomes, and (c) specification and monitoring of policies [28]. Big data offers additional value to the formulation and evaluation of hearing healthcare policy by supplying high-quality evidence [29]. Integrated city data can describe the prevalence and location of housing-related health problems and make housing code enforcement more efficient, effective, and equitable, when responding to public health threats [30]. Sentiment analysis as a data analytics tool helps rapidly and objectively assess the consistency of health communications during a public health crisis [31]. Internet search data can be an additional tool for understanding public opinion about sensitive and/or stigmatizing topics [32]. The emergence of numerous public emergencies in online public opinion offers government officials guidance and proposals on how to control and manage network public opinion [33]. By forecasting the demand trend for essential medicines to treat NCDs, AI can further improve operational management and health supply chain planning [34]. Our findings can help policymakers guide investments toward improving surveillance infrastructure. Multi-national planners can also use these tools to make informed decisions to improve surveillance systems [35].

### Context

AI makes it easier to modify the context of health systems by identifying the precise causes of policy failure, situational awareness, and early crisis warning. Researchers are working on situational awareness for the real-time perception of environmental circumstances in public health situations, using techniques such as data collection, data fusion, and data mining [36]. In order to meet the needs of the public and to realize the public’s demand for subjects, contents, and forms of health literacy service change in some situations, such as the era of COVID-19, one study highlighted the era of big data and the need for targeted public outreach regarding health education and health literacy [37]. AI assists in the identification of innovative economic policy measures that could be implemented prior to an epidemic in the context of macroeconomics [38]. Another study discovered that using AI to explore the social context can reduce decision-making bias and improve communication effectiveness [39].

Furthermore, the public’s bias may cause them to make poor decisions as a result of reading or listening to a message, which can lead to harm or even death. To reduce bias in decision-making, researchers are investigating psychological theories that simulate how bias occurs in PH (Public health) messaging, identifying ways in which PH agencies can apply such strategies to improve the effectiveness of their messages, and examining the range of emotions and sentiments elicited by public health information posts [39, 40]. One study looked at how sociodemographic characteristics affected food labeling and whether it may eventually raise consumer knowledge and consumption [41].

GIS (Geographic information system) can be used as an AI application in public health and other sectors for geospatial data integration, analysis, and visualization of space-time events using web advances. The growing availability of web resources from distributed spatial data portals, global geospatial libraries, and an expanding suite of web integration tools will open up new avenues for disease surveillance, control, and prevention. It will also ensure public access and community empowerment in public health decision-making. New technologies such as data mining and natural language processing will contribute to managing national emergencies, catastrophic events, and risk management [42], which can enhance public trust in the government [43]. To make public health decision-making more intelligent, geo-analytics and intelligence need to use a system thinking approach that leverages big data analysis and other corresponding technologies. This will help develop more effective and targeted interventions [44] or even political issues such as the construction of a national perspective [45].

Other contributions of AI to policymaking that can improve health or minimize its negative effects on other interventions include helping policymakers determine targeted interventions based on habitation-level prediction [42], managing public opinion to prevent social panic, and promoting social harmony and stability [39], or defining place-based thresholds for important environmental features. Finally, thresholds can help public health authorities, engineers, planners, and legislators keep track of current environmental circumstances and healthy behaviors [46].

### Content

Health systems may evolve over time as a result of the availability of AI-based apps to assess the context and results of health-related policies. Several articles have introduced new, improved public health service platforms capable of preserving and transmitting a large number of user data in a network environment, while automatically maintaining system stability and providing excellent social application value. These articles investigated the role of AI in designing, implementing, and analyzing the results of simulated public health and healthcare policy interventions [47, 48]. On the other hand, there are new methods, for instance, to simulate various interventions that intend to control outbreaks [49], support the extraction of actionable knowledge on benefit rules from regulatory healthcare policy text [50], effective and feasible resource scheduling optimization [51] and strategies that can increase the flexibility of political decisions and identify ideal solutions for global health [49]. Other studies that would make it easier to create better policies include those that identify important variables linked to the success of legislation [52], ascertain the impact of policy instruments on targets [53], offer a tool to assess the dispatching procedures for the network of departments [54], and identify variables that can predict behavior [55].

AI also can help determine the optimal allocation of resources based on priorities. New kinds of health financing, the same as tax policy for responding to public health emergencies, are an example of incentives that help quickly respond to public health emergencies [56]. Insurance fund managers can use digital solutions to determine the best allocation of resources based on priorities. In addition, AI leads to a more innovative approach to social inclusivity initiatives in health insurance funds that require a well-defined technology policy to understand the social determinants of health insurance. This can be combined with machine learning algorithms to promote healthy lifestyles and illness prevention techniques in order to improve universal health care [57]. Furthermore, AI assists policymakers in developing policies and investing in infrastructure to scale up exit policies based on risk predictions [58].

AI has the ability to improve lives [57], advance the sustainable development goals (SDGs), and promote health equity [59, 60]. It can assess the availability of public health advice on infectious diseases, which may open up new possibilities for an adaptive statistical formula for a quick assessment of the availability of health advice for those who are most vulnerable [49]. In addition, it can assist multi-sector/multi-actor policy deliberation to select effective policy portfolios [61], anticipate public health interventions from reading news articles [62], and examine policy attitudes [63] that can be applied to other disciplines and alter the structure of the health system. More accountable, responsive, transparent, and citizen-centric government services can be achieved with AI-based apps [64].

### Actors

The beneficiaries’ behavior may be impacted by the emergence of new methods for evaluation. For instance, in order to identify successful policy portfolios that maintain a balance between social, economic, and environmental objectives, policymakers can utilize AI to organize change in complex, non-linear, multi-sector policy contexts [65]. Using Praedico, data can be graphed, mapped, analyzed, and shared with key decision-makers and stakeholders [21] or other platforms monitoring and evaluating a policy, allowing timely responses through policymaker interactions [66]. These methods help public health decision-makers and professionals to create collaborative learning environments that engage beneficiaries [67]. For example, by forecasting the behavior of epidemic outbreaks [68] or promoting health literacy development, we can encourage each citizen to be responsible for their health, guide people to establish a correct health perspective, form a healthy lifestyle, ecological environment, social environment, and prevent public problems caused by diseases before they happen [37]. One study reported that users’ interaction with public health posts increased, while photo and link type posts were the most favorable for high and medium user engagement, respectively [69] These can be used as new methods to increase citizen engagement in health-related topics.

The engagement of stakeholders is critical to the successful formulation and implementation of environmental policies. Also, computational methods can be used to better extract opinions from electronic texts. For example, a study based on a million-word corpus compared stakeholder concerns (i.e., the government (GOV), non-governmental organizations (NGO), and news media) [70]. Another example is the dynamic evaluation of the public’s emotional tendencies; the results of this study revealed differences by sentiment analysis between cities and regions. This can be extended and used as a decision support point for government agencies to avoid misinformation, for example, to promptly adjust policies such as vaccination in response to public health events [71], or subjects related to other communicable and non-communicable diseases and ability to match risk prevention areas and balance resource allocation in the context of community collaborative prevention and control [72]. Based on stakeholder arguments, new recommendations made it possible to reduce the negative impact of some actors on health by targeting specific audiences. By extending Dung’s seminal argumentation system, one study proposed a general model for recommendation-based argumentation. This method is used to examine food quality debates in public health policy. It can make new recommendations based on the arguments of stakeholders by targeting specific audiences [73].

Using the Mining Safety and Health Administration (MSHA) as a new light on the regulatory approach to workplace safety, researchers discussed how, despite using an econometric approach that was designed to overstate the efficacy of MSHA activities, the impact is negligible, and inspection costs continue to outweigh benefits [74]. These investigations can help the community reallocate funding by providing data for exorbitant operational evaluations of health-related issues. Another example is the evaluation of public health advertisements (PHAs) on television that can raise community awareness of the negative effects of social determinants of health, encourage active participation, and advise policymakers on targeted interventions based on these predictions by identifying patterns. Predictive models can also be used to educate the public about the negative effects of social determinants of health and to encourage active participation [75, 76].

### Process

#### Agenda setting

Using digital solutions paves the way for making more evidence-based decisions in the future, which translates into avoiding the refused model. Studies included a discussion on evaluation and early-warning systems based on context awareness for a situational crisis [77] or other environmental problems, such as the influence of air pullution. One study reported a model to assess the potential consequences of air pullution on health and financial losses. The possibility of formulating new strategies, policies, and plans according to the results of forecasting, evaluation, and early warning [78] or predicting and informing public health interventions [79] has a ppotential to transform policymaking. Media coverage to support policy has been the subject of several studies [80]. Hotspot Mining makes it feasible to locate areas with greater needs, choose the best distribution of resources, and create successful intervention programs [81], all of which contribute to better agenda formulation [80]. leading to improved agenda setting. Mining news media to categorize policy interference-related posts [82] and understand public health concerns makes new kinds of information available for decision making. A study found that news coverage of seven public health issues (i.e., “smoking,” “exercise,” “alcohol drinking,” “diet,” “obesity,” “depression,” and “asthma”) has decreased over time. Using big data to address public health priorities is an innovative approach for addressing community needs. Text mining techniques can be used to overcome the limitations of traditional qualitative methodologies [83], and overcome the drawbacks of conventional qualitative methodologies [84]. Risk-based strategies can prioritize high- and medium-risk patients, while lowering healthcare expenses [85]. The ability to match risk prevention areas, balance resource allocation in the context of community collaborative prevention and control [72], prioritize resource allocation based on predictions [86], and innovate ways to represent the voices of structurally vulnerable groups [87] are other examples that can change agenda setting situations.

#### Implementation

Through new capabilities such as optimal policy learning, AI can assist in the resolution of problems such as refusal to participate. One study proposed that future air pollution policymaking may pay more attention to pressing concerns about its health consequences and financial burden, with health experts becoming more closely involved in regulatory decision-making [88]. New ways of resource allocation [89], estimating the effectiveness of interventions, simulating indifferent transmission scenarios [90], and routine evaluation of policies, interventions, and indicators will provide new opportunities for implementing policies, which may be ignored in the usual situation. Evaluating the effectiveness of strategies before and after implementation can help policymakers decide about policy changes and avoid the cost of incorrect decisions [91]. On the other hand, new analytics provide a vehicle to measure the effectiveness of such overarching strategies and policies [91, 92], and enables policymakers to simulate future scenarios in response to specific health conditions [92]. Researchers discussed how grocery transaction data with price, discounting, and other product attributes provide an opportunity to evaluate the likely effects of taxation policy. The findings revealed that sales are non-linearly related to price and are influenced by the prices of multiple competing brands. They also demonstrated how machine learning methods applied to large amounts of transactional data from supermarkets can provide evidence that may assist guiding public health policy [93]. In another study, researchers used three analytical techniques—two econometric and one machine learning—to evaluate the effect of the subsidy on the demand for organic fruit in representative middle-class, wealthy, and poor US households. Other studies that come to the conclusion that the use of models and algorithms acting as critical instruments to bridge the gap between research and policy [94], include the prediction of cost and the regulation of tax policies, both of which can help to combat catastrophes such as the pandemic [22].

Overwhelming preliminary evidence shows that the analysis of AI related to the effects of policies [95] or simulation of exit strategies for health issues such as pandemics [96] and NCD, will alter the process of policymaking in the future.

#### Evaluation

AI has the potential to monitor policy changes and evaluate their impact and risk on a particular population, which can remove various societal, cultural, regulatory, normative, governance, and policy constraints [26]. Therefore, predicting health outcomes and providing policy information, policy analysis [97], policy empowerment [26], space-specific ‘health checks,’ and assessments [98] are some of the AI’s new capabilities in the evaluation of health systems. Policymakers can devise control measures to address future threats to public health with the use of functional assessments, measurements of value, and assessments of efficacy [21, 99]. In addition, public health policymaking can utilize all available and quickly developing data sources [26], such as by assessing risk factors, to produce surveillance data and help with the adoption, implementation, and enforcement of policies [100]. Platforms that support the evaluation of public health policy [66] make policy recommendations and design public health interventions at the municipal or other jurisdictional scales [100].

Estimating the various policy effects associated with modifications is another application of AI [101] One study used the Korea Medical Insurance Corporation database to show how knowledge discovery and data mining algorithms can predict health outcomes and provide policy information for hypertension management. The CHIAD algorithm and the association rule also provided segment-specific data for the risk variables and target group, which could be used in policy analysis for hypertension management [97].

Large-scale automated analysis of news media can be accomplished using AI. One study, for example, categorizes obesity as an individual-level problem (e.g., diet) and/or an environmental-level problem (e.g., obesogenic environment) [102]. In another study, researchers presented an Epidemic Sentiment Monitoring System (ESMOS) that provides tools for visualizing Twitter users’ concerns about various diseases, by developing a novel two-step sentiment classification workflow to automatically identify personal tweets and negative tweets [103]. In recent years, there has been significant interests in researching methods to detect favorable and unfavorable attitudes toward specific subjects, such as public health opinions [104]. Despite its advantages, this research had some limitations. Our exploratory study of AI applications in health policy was broad rather than specific. Additional research is required to bridge the gaps, e.g., the biases in computer programs raise both ethical and practical concerns. Although AI tools ultimately make health policy more manageable, trust is necessary at different points in the process to prevent inequalities. Therefore, future research should focus on the need for open, accountable, and equitable AI technologies and applications.

## Conclusion

When applied to complex issues such as health systems, traditional policymaking tools face serious limitations, particularly in recent years with ongoing emergencies (e.g., outbreaks or pandemics). AI solutions prove to be more sophisticated and interconnected as they spread throughout healthcare systems. As a result, health systems need to invest more in AI to design policies and make better decisions in order to save more lives. Our research introduced two frameworks (Figs. 2 and 3) that can be used in future studies to prevent the negative effects of contextual factors on health systems. We advocate the use of these frameworks to develop more effective, efficient and tailored health policies.

### Electronic supplementary material

Below is the link to the electronic supplementary material.

Supplementary Material 1

## Data Availability

All data generated or analyzed during this study are included in this published article and its supplementary information files.

## Abbreviations

AI: Artificial intelligence
IoT: Internet of things
NCDs: Non-communicable Diseases
PDT: Policy Development Toolkit
HiAP: Health in All Policy Making
PH: Public health
MIDAS: Meaningful Integration of Data Analytics and Services
GIS: Geographic information system
SDGs: Sustainable Development Goals
MSHA: Mining Safety and Health Administration
GOV: Government
NGO: Non-Governmental Organization (NGO)
MEDIA: News media
PHAs: Public Health Announcements
ESMOS: Epidemic Sentiment Monitoring System
