# Supplementary material for: The application of artificial intelligence in health policy: a scoping review
Source: BMC Health Serv Res. 2023 Dec 15;23:1416. doi: 10.1186/s12913-023-10462-2 (PMC10722786; doi:10.1186/s12913-023-10462-2)
Supplement: Supplementary file 1 — Supplementary Material 1 [file 12913_2023_10462_MOESM1_ESM.docx]

Ramezani et al. (2023)

Supplementary File 1

Appendix 1: details of included studies

As applications and capabilities of AI contribute to elements of policy triangulate. Framework 1 (on the main text of Article) are developed as an agenda for future studies using the findings in table 1.

**Table 1. Summary of included studies**

|  | **First Author/year** | **Title** | **Main result** | **Interaction with context** |
| --- | --- | --- | --- | --- |
| 1 | Jagrič (2022) | Reshaping the Healthcare Sector with Economic Policy Measures Based on COVID-19 Epidemic Severity: A Global Study. | identify the areas where the application of economic policy measures would enhance the resilience of societies on epidemic risks[1] | artificial intelligence  context: situational, economical  content: innovative identification matrix based interventions  process: agenda setting (advocacy) |
| 2 | Wen (2022) | A dynamic machine learning model to predict and inform non-pharmacological public health interventions from global news reports | predict and inform non-pharmacological public health interventions from global news reports  [2] | machine learning  context: situational, legal, technical  content: news reports based interventions  process: agenda setting |
| 3 | Bulut (2022) | Rapid assessment of communication consistency: sentiment analysis of public health briefings during the COVID-19 pandemic | practical implications and methodological advantages of using sentiment analysis as a data analytics tool for rapidly and objectively assessing the consistency of health communications during a public health crisis [3] | machine learning  context: social, situational, legal  content: sentiment analysis based policies  process: agenda setting, implementation, evaluation |
| 4 | Martin-Moreno (2022) | Predictive Models for Forecasting Public Health Scenarios: Practical Experiences Applied during the First Wave of the COVID-19 Pandemic | Forecasting the behavior of epidemic outbreaks [4] | machine learning  context: technical, situational, legal  content; Forecasting based policies  process: agenda setting |
| 5 | Gauld (2022) | Popular and Scientific Discourse on Autism: Representational Cross-Cultural Analysis of Epistemic Communities to Inform Policy and Practice | inform policy makers on the health and social needs and concerns of individuals and their caregivers, especially to define health indicators based on important issues for beneficiaries [5] | Machine learning  context: social, political, legal  content; need and concern based policies  process: agenda setting, evaluation |
| 6 | Varela (2022) | Prediction of SARS-CoV-2 infection with a Symptoms-Based model to aid public health decision making in Latin America and other low and middle income settings | prioritize resource allocation related to COVID-19 diagnosis, to decide on early isolation, and contact-tracing strategies in individuals [6] | machine learning  context: social, situational, legal  content; Disease Prevention based policies  process: agenda setting |
| 7 | Nath (2022) | Predicting the distribution of arsenic in groundwater by machine learning technique in two worst hit districts of Assam, India: a risk to public health | The habitation-level predictive model can be used to inform villagers and generate community awareness about the potential impact of elevated As in groundwater through active participation.  advise policymakers on targeted interventions related to the habitation-level predictive [7] | Machine Learning  context: social, Enviorenmental , legal  content: habitation-level predictive policies  process: agenda setting, evaluation |
| 8 | Hao (2022) | Deep Q networks-based optimization of emergency resource scheduling for urban public health events | optimizes and upgrades the urban emergency resource scheduling scheme mainly from the aspect of path planning for the distribution of emergency resources  The system simulation experiment proves that the urban emergency resource scheduling optimization scheme based on deep learning is effective and feasible.  [8] | Big data  Context: Technical, situational, international factor (pandemic)  Content: resource scheduling optimization  Process: agenda setting, Implementation |
| 9 | Chen (2021) | Efficient Social Distancing during the COVID-19 Pandemic: Integrating Economic and Public Health Considerations | allows us to simulate possible future scenarios in response to specific mobility indices and thus verify the effectiveness of social distancing policies [9] | Deep learning  Content: simulate the effectiveness of policies  context: legal, situational  process: evaluation |
| 10 | Zhong (2022) | Metamodeling for Policy Simulations with Multivariate Outcomes | Metamodeling is an important tool for making results from complex models accessible to decision makers. This study provides a framework for metamodeling in policy analyses with multivariate outcomes[10] | Content: metamodeling evaluation based policies  context: legal, technical  process: evaluation |
| 11 | Oselio (2022) | , B., et al., Reinforcement learning evaluation of treatment policies for patients with hepatitis C virus | Off-policy evaluation strategies are useful to evaluate hypothetical treatment policies without implementation. If a quality risk model is available, risk-based treatment strategies can reduce overall risk and prioritize patients while reducing healthcare system costs [11] | Big Data  Content: risk based policies  Context: technical, legal, economical  Process: agenda setting |
| 12 | Tian (2022) | Improvement of the Public Health Service Platform System Based on the Big Data-Driven System | improved public health service platform system can store and transmit a large number of user data in the network environment, automatically maintaining the stability of the system and has a good social application value [12]. | Big Data  Content: platform based policies  Context: technical  Process: agenda setting, implementation, evalution |
| 13 | Farkhad (2022) | State health policies and interest in PrEP: evidence from Google Trends | internet search data can be used as an additional tool for understanding public opinion about sensitive and/or stigmatizing topics  [13] | Machine Learning  Content: opinion-based policies  Context: technical  Process: agenda setting |
| 14 | Xu (2021) | Digital Surveillance to Identify California Alternative and Emerging Tobacco Industry Policy Influence and Mobilization on Facebook | Posts were manually annotated to characterize themes associated with industry political interference and user interaction.  categories of policy interferencerelated posts [14] | data mining  Content: social media-based policies  Context: technical  Process: agenda setting, imolementation, evaluation |
| 15 | Espinosa (2022) | Epitweetr: Early warning of public health threats using Twitter data | early detection of public health threats using Twitter data[15] | Machine Learning  Content: social media-based policies  Context: technical, situational  Process: agenda setting |
| 16 | Pathak (2021) | Sentiments Evoked by WHO Public Health Posts During the COVID-19 Pandemic: A Neural Network-Based Machine Learning Analysis | explores the range of emotions and sentiments evoked by public health information posts [16] | Machine Learning  Content: social users' concern-based policies  Context: social, political, situational  Process: agenda setting, implementation |
| 17 | Zhang (2021) | The Impact of Public Health Events on COVID-19 Vaccine Hesitancy on Chinese Social Media: National Infoveillance Study. | Analyze and change the positive and negative tendencies exist with respect to expressed emotional tendencies by the public.  willingness to accept a vaccine through the use of positive incentives with prompt responses to pandemic-related news [17] | Big data  Content: social users' concern-based policies  Context: social, political, situational  Process: implementation |
| 18 | Buchbinder (2022) | Advancing a Data Justice Framework for Public Health Surveillance | innovate ways to represent the voices of structurally vulnerable groups in the design and governance of big data initiatives [18] | big data  Content: surveilance-based policies  Context: technical  Process: agenda setting |
| 19 | Prasinos (2022) | A Modelling Framework for Evidence-Based Public Health Policy Making | an integrated platform for analysis of heterogeneous data, including health care devices usage, physiological, cognitive, clinical and medication, personal, behavioural, lifestyle data, occupational and environmental data [19] | big data  Content: evidence-based policies  Context: technical  Process: agenda setting |
| 20 | Zhang (2022) | Public Health Risk Assessment and Prevention Based on Big Data | ability of matching risk prevention areas and balancing resource allocation in the context of community collaborative prevention and control [20] | Content: need based interventions  Context: social factor, political, economical  Process: evaluation |
| 21 | Nijkamp (2022) | Place-Specific Corona Dashboards for Health Policy: Design and Application of a ‘Dutchboard’ | space-specific ‘health checks’ and assessments [21] | Content: Place-Specific Dashboards based policies  Context: technical, situationsl  Process: agenda setting, evaluation |
| 22 | Zhu (2022) | Can artificial intelligence enable the government to respond more effectively to major public health emergencies?——Taking the prevention and control of Covid-19 in China as an example | effectively solve and prevent the further development of the situation, and at the same time improve the government's ability and level to respond to major public health emergencies, and increase the government's prestige in the eyes of the public.  control the correct orientation of public opinion  avoid social panic and affect social harmony and stability [22] | Content: need based interventions  Context: social factor  Process: evaluation |
| 23 | Asheer (2022) | Balancing national economic policy outcomes for sustainable development | enable policymakers to sift through complex, non-linear, multi-sector policy spaces to identify efficient policy portfolios that balance economic, social, and environmental goals.  support multi-sector, multiactor policy deliberation to screen efficient policy portfolios[23] | Content: efficient policy portfolios  Context: technical, economical, social, enviornmental  Process: agenda setting, evaluation |
| 24 | Shi (2022) | System Architecture of a European Platform for Health Policy Decision Making: MIDAS | The MIDAS platform delivers a secure, effective and integrated solution to deal with health data, providing support for health policy decision-making, planning of public health activities and the implementation of the Health in All Policies approach [24] | Content: Health in All Policies approach  Context: technical, legal, social  Process: agenda setting, evaluation |
| 25 | Evgeniou (2022) | Pandemic lockdown, isolation, and exit policies based on machine learning predictions | develop policies and invest in infrastructure to operationalize personalized isolation and exit policies based on risk predictions at scale [25] | machine learning and artificial intelligence  Content: risk-based policies  Context: technical, legal factor  Process: agenda setting, evaluation |
| 26 | Zhang (2022) | The Construction of National Image of China by English World Media in Public Health Emergencies | Construction of National Image [26] | Data mining  Content: opinion-based policies  Context: political factor  Process: evaluation |
| 27 | Zhang (2022) | Public View of Public Health Emergencies Based on Artificial Intelligence Data | in-depth analysis of the evolution process of online public opinion on public emergencies from the macro-, meso-, and micro-perspectives, in order to analyze the dissemination methods and internal evolution mechanism of various public emergencies of online public opinion, which provides countermeasures and suggestions for the government to guide and manage network public opinion[27] | Artificial Intelligence  Content: public opinion based interventions  Context: social, political, technical, situational  Process: evaluation |
| 28 | Wu (2022) | Assessment of the Benefits of Targeted Interventions for Pandemic Control in China Based on Machine Learning Method and Web Service for COVID-19 Policy Simulation | estimate  the effectiveness  of  interventions  and  simulate transmission  in  different  scenarios [28] | machine learning  Content: estimation-based policies  Context: legal factor, technical  Process: evaluation |
| 29 | Mbonyinshuti (2022) | Application of random forest model to predict the demand of essential medicines for non-communicable diseases management in public health facilities. | predict demand trends and global prevalence of Non-Communicable Diseases (NCD) and the economic and clinical burden they impose  optimize health supply chain planning and operational management by boosting the accuracy in predicting the demand trend for NCD essential medicines [29] | machine learning  Content: prediction-based policies  Context: legal factor  Process: evaluation |
| 30 | Ramírez (2022) | Prediction of SARS-CoV-2 infection with a Symptoms-Based model to aid public health decision making in Latin America and other low and middle income settings. | prioritize resource allocation based on predictions [30] | machine learning  Content: priority-based analysis  Context: political and situational, legal factor  Process: agenda setting, evaluation |
| 31 | Picco (2022) | Learning Insurance Benefit Rules from Policy Texts with Small Labeled Data. | support the extraction of actionable knowledge on benefit rules from regulatory healthcare policy text [31] | machine learning  Content: rule-based analysis  Context: legal factor  Process: agenda setting |
| 32 | Wang (2022) | From policy to prediction: Forecasting COVID-19 dynamics under imperfect vaccination. | help policymakers design control measures as variant strains threaten public health [32] | machine learning  Content: policy-based analysis  Context: political and situational, legal factor  Process: evaluation |
| 33 | Gao (2022) | How to improve public health literacy based on polycentric public goods theory: preferences of the Chinese general population. | Assist the public in self-management of their health.  publicize health education and health literacy in a targeted way, to meet the public’s needs, and to understand how the public’s demand for subjects, contents, and forms of health literacy service has changed [33] | Content: need based interventions  Context: social factor  Process: evaluation |
| 34 | Basheer (2022) | Balancing national economic policy outcomes for sustainable development. | identifying policy portfolios that achieve efficient mixes of poverty and inequality reduction, economic growth, and climate change mitigation.  determine the relative influence of policy instruments on sustainability targets [34] | Big data  Context: situational, international factor (pandemic)  Content: context-based guidelines  Process: agenda setting, policy learning |
| 35 | Lucero‑Obusan (2022) | Public health surveillance in the US Department of Veterans Affairs: evaluation of the Praedico surveillance system | Praedico is a customizable surveillance and data analytics platform built on big data technologies. Functionality is straightforward, with rapid query generation and runtimes. Data can be graphed, mapped, analyzed, and shared with key decision makers and stakeholders.  future system evaluations include measurements of value and efectiveness along with additional organizations and functional assessments[35] | Big data  Context: situational, international factor (pandemic)  Content: context-based guidelines  Process: agenda setting, policy learning |
| 36 | Zhi Wen (2022) | [Inferring global-scale temporal latent topics from news reports to predict public health interventions for COVID-19](https://www.scopus.com/record/display.uri?eid=2-s2.0-85126070550&origin=resultslist&sort=plf-f&src=s&nlo=&nlr=&nls=&sid=0512b12e36be5e7ef97b213d5fcab6ea&sot=a&sdt=a&sl=534&s=%28+%28+TITLE+%28+%22policy%22+%29+OR+TITLE+%28+%22global+health%22+%29+OR+TITLE+%28+%22public+health%22+%29+OR+TITLE+%28+%22international+health%22+%29+OR+TITLE+%28+%22policies%22+%29+OR+TITLE+%28+%22national+reform%22+%29+OR+TITLE+%28+%22priority+setting%22+%29+OR+TITLE+%28+%22national+program%22+%29+OR+TITLE+%28+%22public+administration%22+%29+OR+TITLE+%28+%22national+strategy%22+%29+%29+%29+AND+%28+TITLE+%28+health+%29+%29+AND+%28+%28+TITLE-ABS-KEY+%28+%22big+data%22+%29+OR+TITLE-ABS-KEY+%28+%22data+mining%22+%29+OR+TITLE-ABS-KEY+%28+%22artificial+intelligence%22+%29+OR+TITLE-ABS-KEY+%28+%22deep+learning%22+%29+OR+TITLE-ABS-KEY+%28+%22machine+learning%22+%29+%29+%29&relpos=9&citeCnt=0&searchTerm=) | predict public health interventions.  latent topics from news reports to predict public health interventions[36] | Machine learning  Context: situational, international factor (pandemic)  Content: context-based guidelines  Process: agenda setting, policy learning |
| 37 | Darren J. Edwards (20210 | [Ensuring Effective Public Health Communication: Insights and Modeling Efforts From Theories of Behavioral Economics, Heuristics, and Behavioral Analysis for Decision Making Under Risk](https://www.scopus.com/record/display.uri?eid=2-s2.0-85118191560&origin=resultslist&sort=plf-f&src=s&nlo=&nlr=&nls=&sid=0512b12e36be5e7ef97b213d5fcab6ea&sot=a&sdt=a&sl=534&s=%28+%28+TITLE+%28+%22policy%22+%29+OR+TITLE+%28+%22global+health%22+%29+OR+TITLE+%28+%22public+health%22+%29+OR+TITLE+%28+%22international+health%22+%29+OR+TITLE+%28+%22policies%22+%29+OR+TITLE+%28+%22national+reform%22+%29+OR+TITLE+%28+%22priority+setting%22+%29+OR+TITLE+%28+%22national+program%22+%29+OR+TITLE+%28+%22public+administration%22+%29+OR+TITLE+%28+%22national+strategy%22+%29+%29+%29+AND+%28+TITLE+%28+health+%29+%29+AND+%28+%28+TITLE-ABS-KEY+%28+%22big+data%22+%29+OR+TITLE-ABS-KEY+%28+%22data+mining%22+%29+OR+TITLE-ABS-KEY+%28+%22artificial+intelligence%22+%29+OR+TITLE-ABS-KEY+%28+%22deep+learning%22+%29+OR+TITLE-ABS-KEY+%28+%22machine+learning%22+%29+%29+%29&relpos=72&citeCnt=1&searchTerm=) | improve the effectiveness of messages.  minimize the problem of bias in decision making and improve the effectiveness of messages [37] | Machine learning  Context: economic, social, political  Content: cognitively bias and PH messages-based policies  Process: agenda setting, implementation |
| 38 | THOMAS J. KNIESNER (2004) | [Data mining: MSHA enforcement efforts, underground coal mine safety, and new health policy implications](https://www.scopus.com/record/display.uri?eid=2-s2.0-4444364116&origin=resultslist&sort=plf-f&src=s&sid=02e2386f03135aa96570babb26477976&sot=a&sdt=a&sessionSearchId=02e2386f03135aa96570babb26477976&relpos=252&citeCnt=26) | the regulatory approach to workplace safety for  cost per life, cost-effectiveness, value of life, life years  reallocating budget.  Mine Safety and Health Administration (MSHA) as a new light on the regulatory approach to workplace safety[38] | Data mining  Content: new health policy implication-based policies  Context: legal, social, environment factor  Process: agenda setting, evaluation |
| 39 | Katharine Robb (2022) | [Using Integrated City Data and Machine Learning to Identify and Intervene Early on Housing-Related Public Health Problems](https://www.scopus.com/record/display.uri?eid=2-s2.0-85107849561&origin=resultslist&sort=plf-f&src=s&nlo=&nlr=&nls=&sid=0512b12e36be5e7ef97b213d5fcab6ea&sot=a&sdt=a&sl=534&s=%28+%28+TITLE+%28+%22policy%22+%29+OR+TITLE+%28+%22global+health%22+%29+OR+TITLE+%28+%22public+health%22+%29+OR+TITLE+%28+%22international+health%22+%29+OR+TITLE+%28+%22policies%22+%29+OR+TITLE+%28+%22national+reform%22+%29+OR+TITLE+%28+%22priority+setting%22+%29+OR+TITLE+%28+%22national+program%22+%29+OR+TITLE+%28+%22public+administration%22+%29+OR+TITLE+%28+%22national+strategy%22+%29+%29+%29+AND+%28+TITLE+%28+health+%29+%29+AND+%28+%28+TITLE-ABS-KEY+%28+%22big+data%22+%29+OR+TITLE-ABS-KEY+%28+%22data+mining%22+%29+OR+TITLE-ABS-KEY+%28+%22artificial+intelligence%22+%29+OR+TITLE-ABS-KEY+%28+%22deep+learning%22+%29+OR+TITLE-ABS-KEY+%28+%22machine+learning%22+%29+%29+%29&relpos=16&citeCnt=1&searchTerm=) | Identify and Intervene Early on Housing-Related Public Health Problems location of housing-. related health problems[39] | machine learning  content: location of housing-related health risks  context: social, legal, environment  process: agenda setting |
| 40 | Ilias moglogiannis (2020) | Evidence-based Public Health Policy Models Development and Evaluation using Big Data Analytics and Web Technologies | The Policy Development Toolkit.  cloud-based Decision Support System for public health decision-making[40] | Big Data  Process: (agenda setting) and evaluation  Context: technical factor, legal  Content: Key Performance Indexes (KPIs) based policies |
| 41 | Argyro Mavrogiorgou (2020) | [CrowdHEALTH: An e-Health big data driven platform towards public health policies](https://www.scopus.com/record/display.uri?eid=2-s2.0-85091464715&origin=resultslist&sort=plf-f&src=s&sid=41e6799fd5bcf297cdff81e2a617e7d9&sot=a&sdt=a&sessionSearchId=41e6799fd5bcf297cdff81e2a617e7d9&relpos=44&citeCnt=0) | platform towards health in all policies[41] | Big data  Content: health determinants-based policies  Context: legal, social, environment factor  Process: agenda setting |
| 42 | Dimosthenis Kyriazis (2019) | [The CrowdHEALTH project and the hollistic health records: Collective wisdom driving public health policies](https://www.scopus.com/record/display.uri?eid=2-s2.0-85085931503&origin=resultslist&sort=plf-f&src=s&sid=072d64c81ec0fbf627c17ba1506d44ca&sot=a&sdt=a&sessionSearchId=072d64c81ec0fbf627c17ba1506d44ca&relpos=57&citeCnt=2) | provision of insight for different population segments according to different factors.  capturing all health determinants in the proposed HHRs (Holistic Health Records) [42] | big data  content: different population segment-based policies  context: environment factor (e.g. location, occupation, medication status, emerging risks, etc), legal  process: agenda setting |
| 43 | Alice G (2020) | Health in All Policy Making Utilizing Big Data | Causal Analysis,  Forecasting, and Risk Stratification.  the Policy Development Toolkit (PDT) [43] | Big Data  Content: integrated policies and need based policies  Process: agenda setting  Context: legal, political |
| 44 | KBioAssist, S (2017) | Crowd HEALTH: Holistic Health Records and Big Data Analytics for Health Policy Making and Personalized Health | Cross-domain co-creation of policies  new paradigm of Holistic Health Records (HHRs) [44] | Big Data  Content: integrated based policies  Context: legal, political, environmental factor  Process: agenda setting, evaluation |
| 45 | Brian Cleland (2019) | [Usability Evaluation of a Co-created Big Data Analytics Platform for Health Policy-Making](https://www.scopus.com/record/display.uri?eid=2-s2.0-85069809567&origin=resultslist&sort=plf-f&src=s&sid=b20965c6ba11a73b8bef379611d7eeb9&sot=a&sdt=a&sessionSearchId=b20965c6ba11a73b8bef379611d7eeb9&relpos=98&citeCnt=2) | Platform based policies  MIDAS platform[45] | Big Data  Context: technical factor, international factor  process: agenda setting  content: Platform based policies  process: agenda setting |
| 46 | ZHOU Jing (2015) | Framework Construction and Application for Global Health Information Platform | Global Health Information Platform  [46] | big data  Content: Platform -based policies  Context: international, situational, social, legal  Process: agenda setting |
| 47 | Konstantinos Moutselos (2020) | [A Web based modular environment for assisting health policy making utilizing big data analytics](https://www.scopus.com/record/display.uri?eid=2-s2.0-85062824679&origin=resultslist&sort=plf-f&src=s&sid=b20965c6ba11a73b8bef379611d7eeb9&sot=a&sdt=a&sessionSearchId=b20965c6ba11a73b8bef379611d7eeb9&relpos=114&citeCnt=3) | forecasting, causal and risk analysis, as well for the compilation and visualization of predictions.  Policy Development Toolkit[47] | Big data  Content: holistic health related policies  Context: social factor (problem of the aging society), technical  Process: agenda setting, implementation, evaluation, platform |
| 48 | Spanoudakis, G (2017) | Public health policy for management of hearing impairments based on big data analytics: EVOTION at Genesis | integrated platform project. A project to develop integrated platform supporting  EVOTION (for management of hearing impairments) [48] | big data  Content: integrate based policies  Context: social, technical  Process: agenda setting, evaluation |
| 49 | Iain Buchan (2010) | IMPACT: A generalisable system for simulating public health interventions | simulating public health interventions. A system for analyzing the results of healthcare policy interventions[49] | Machine learning  Content: interventions simulating based policies  context: legal, political  process: agenda setting, evaluation |
| 50 | Marco Anisetti (2017) | Big data platform for public health policies | supporting automatically monitoring and evaluation of a given policy & timely response allowing interactions with policy makers. A platform supporting the evaluation of Public Health Policy[50] | Big data  Content: evidence-based analysis-based policies  Context: political (interactions with policy makers) and technical, legal factor  Process: agenda setting (politics stream), evaluation |
| 51 | J. D. Ainsworth (2011) | IMPACT: a generic tool for modelling and simulating public health policy | Discrete event simulation- the ability to connect health policy-modelers and policy-makers in a unified system, thereby making population health models easier to share, maintain, reuse and deploy. A Tool for Modelling and Simulating Public Health Policy[51] | Content: simulated based policies  Context: legal, political, technical  Process: agenda setting, evaluation |
| 52 | Health Unit (2008) | Strategies to improve global influenza surveillance: A decision tool for policy-makers | help improve surveillance.  decision tool for policy-makers[52] | data mining  Context: surveillance-based policies  Context: social, political, legal, situational  Process: agenda setting (financial feasibility) |
| 53 | Yingzhi Li (2022) | [Research on public health crisis early warning system based on context awareness](https://www.scopus.com/record/display.uri?eid=2-s2.0-85126072379&origin=resultslist&sort=plf-f&src=s&nlo=&nlr=&nls=&sid=0512b12e36be5e7ef97b213d5fcab6ea&sot=a&sdt=a&sl=534&s=%28+%28+TITLE+%28+%22policy%22+%29+OR+TITLE+%28+%22global+health%22+%29+OR+TITLE+%28+%22public+health%22+%29+OR+TITLE+%28+%22international+health%22+%29+OR+TITLE+%28+%22policies%22+%29+OR+TITLE+%28+%22national+reform%22+%29+OR+TITLE+%28+%22priority+setting%22+%29+OR+TITLE+%28+%22national+program%22+%29+OR+TITLE+%28+%22public+administration%22+%29+OR+TITLE+%28+%22national+strategy%22+%29+%29+%29+AND+%28+TITLE+%28+health+%29+%29+AND+%28+%28+TITLE-ABS-KEY+%28+%22big+data%22+%29+OR+TITLE-ABS-KEY+%28+%22data+mining%22+%29+OR+TITLE-ABS-KEY+%28+%22artificial+intelligence%22+%29+OR+TITLE-ABS-KEY+%28+%22deep+learning%22+%29+OR+TITLE-ABS-KEY+%28+%22machine+learning%22+%29+%29+%29&relpos=36&citeCnt=0&searchTerm=) | early warning system based on context awareness. situational awareness and crisis early warning[53] | data mining  Context: situational  Content: situational awareness-based policies  Process: agenda setting, platform |
| 54 | Gabrielle H (2020) | Application of Big Data to Support Evidence-Based Public Health Policy Decision-Making for Hearing | formulate and evaluate the impact of health policies. platform and a synthetic dataset to model the estimated risk of noise induced hearing loss[54] | Big Data  Content: Evidence-Based policies  Context: social  Process: Formulation and evaluation, platform |
| 55 | Hamed M (2018) | [The strategic value of big data analytics in health care policy-making](https://www.scopus.com/record/display.uri?eid=2-s2.0-85048449972&origin=resultslist&sort=plf-f&src=s&sid=de32465af2b2ceb898a62458c65b559d&sot=a&sdt=a&sessionSearchId=de32465af2b2ceb898a62458c65b559d&relpos=117&citeCnt=0) | the feedback can be used to obtain a better understanding of loopholes and to propose more effective policies in prospective endeavors. Evaluate effectiveness of strategies before and after implementation[55] | Big data  Process: evaluation  Context: political factor  Content: strategy evaluation-based policies |
| 56 | Xing Han Lu (2019) | [Guiding Public Health Policy by Using Grocery Transaction Data to Predict Demand for Unhealthy Beverages](https://www.scopus.com/record/display.uri?eid=2-s2.0-85070550516&origin=resultslist&sort=plf-f&src=s&sid=41e6799fd5bcf297cdff81e2a617e7d9&sot=a&sdt=a&sessionSearchId=41e6799fd5bcf297cdff81e2a617e7d9&relpos=45&citeCnt=2) | provide evidence to guide public health policy. evaluate the likely effects of taxation policy[56] | machine learning  Content: Demand for Unhealthy Beverages, effects of taxation policy and Grocery transaction-based policies  Context: social, legal, economic  Process: agenda setting |
| 57 | Ned English (2021) | [Image processing for public health surveillance of tobacco point-of-sale advertising: Machine learning-based methodology](https://www.scopus.com/record/display.uri?eid=2-s2.0-85114053681&origin=resultslist&sort=plf-f&src=s&nlo=&nlr=&nls=&sid=0512b12e36be5e7ef97b213d5fcab6ea&sot=a&sdt=a&sl=534&s=%28+%28+TITLE+%28+%22policy%22+%29+OR+TITLE+%28+%22global+health%22+%29+OR+TITLE+%28+%22public+health%22+%29+OR+TITLE+%28+%22international+health%22+%29+OR+TITLE+%28+%22policies%22+%29+OR+TITLE+%28+%22national+reform%22+%29+OR+TITLE+%28+%22priority+setting%22+%29+OR+TITLE+%28+%22national+program%22+%29+OR+TITLE+%28+%22public+administration%22+%29+OR+TITLE+%28+%22national+strategy%22+%29+%29+%29+AND+%28+TITLE+%28+health+%29+%29+AND+%28+%28+TITLE-ABS-KEY+%28+%22big+data%22+%29+OR+TITLE-ABS-KEY+%28+%22data+mining%22+%29+OR+TITLE-ABS-KEY+%28+%22artificial+intelligence%22+%29+OR+TITLE-ABS-KEY+%28+%22deep+learning%22+%29+OR+TITLE-ABS-KEY+%28+%22machine+learning%22+%29+%29+%29&relpos=93&citeCnt=0&searchTerm=) | novel methodological approach in the collection and processing of data (logo locations) for tobacco or other POS surveillance efforts.  Evaluation of advertising in tobacco point-of-sale[57] | machine learning  context: social factors, political  content: point-of-sale advertising-based policies  process: inform policy adoption, implementation, and enforcement. |
| 58 | Wendong Yang (2021) | A novel framework for forecasting, evaluation and early-warning for the influence of PM10 on public health | health economic losses evaluation model. evaluation and early-warning for the influence of PM10[58] | Artificial intelligence  Content: early-warning and forecasting based policies  Context: environment factor  Process: agenda setting and (forecasting) evaluation |
| 59 | Heui Sug Jo (2019) | [Evaluation of food labeling policy in Korea: Analyzing the community health survey 2014-2017](https://www.scopus.com/record/display.uri?eid=2-s2.0-85071166992&origin=resultslist&sort=plf-f&src=s&sid=6caf27bc704c4753b86461f57cef3ee3&sot=a&sdt=a&sessionSearchId=6caf27bc704c4753b86461f57cef3ee3&relpos=101&citeCnt=1) | develop strategies for community-based education programs and promote the utilization of food labeling. examine the effects of sociodemographic factors on the use of food labeling[59] | data mining  content: dietary lifestyles  based policies  context: social, legal  process: agenda setting |
| 60 | Rita Hamad (2015) | Large-scale automated analysis of news media: a novel computational method for obesity policy research | Large-Scale Automated Analysis of News Media[60] | machine learning  Content: automated analysis of news media-based policies  Context: environment, legal, political, technical  Process: agenda setting, implementation. evaluation |
| 61 | Noemi Kreif (2021) | Estimating heterogeneous policy impacts using causal machine learning: a case study of health insurance reform in Indonesia | re-design of the eligibility criteria for policies.  Estimating heterogeneous policy impacts of health insurance reform[61] | machine learning  Process: policy evaluation, agenda setting  content: heterogeneous -based policies  Context: legal, political |
| 62 | Xiang Ji (2013) | Monitoring Public Health Concerns Using Twitter Sentiment Classifications | Monitoring Public Health Concerns [62] | Machine Learning  Content: social users' concern-based policies  Context: social, political, environment  Process: agenda setting (support) |
| 63 | Arash Barfar (2021) | Pattern discovery, validation, and online experiments: a methodology for discovering television shows for public health announcements | Pattern discovery.  Evaluation of Public Health Announcements (PHAs) on television[63] | big data  content: awareness about risk behaviors and chronic conditions-based policies  context: social, legal  process: agenda setting, implementation |
| 64 | Wenxiu Xie (2021) | Developing Machine Learning and Statistical Tools to Evaluate the Accessibility of Public Health Advice on Infectious Diseases among Vulnerable People | Evaluate the Accessibility of Public Health Advice on Infectious Diseases[64] | machine learning  context: situational  content: Health Advice and equity-based policies  process: agenda setting, implementation, evaluation |
| 65 | G. Lamprell a (2017) | Mainstreaming gender and promoting intersectionality in Papua New Guinea's health policy: a triangulated analysis applying data-mining and content analytic techniques | analyses the policy attitudes toward gender in PNG's health sector[65] | data-mining  Content: policy attitudes and gender equity-based policies  Context: social, political (technocratically), legal  Process: agenda setting |
| 66 | Young Moon Chae  (2001) | Data mining approach to policy analysis in a health insurance domain | predict health outcomes and provide information for  policy analysis. policy analysis in a health insurance domain[66] | Data mining  Content: policy information and segment-specific information-based policies  Context: legal, social, political, technical  Process: agenda setting |
| 67 | Behram Wali (2020) | Developing policy thresholds for objectively measured environmental features to support active travel | Thresholds for key environmental features[67] | machine-learning  Content: environmental conditions, thresholds and healthy behaviors-based policies  Context: environmental factor  Process: agenda setting, implementation |
| 68 | Maged N (2021) | Smart city lifestyle sensing, big data, geo-analytics and intelligence for smarter public health decision-making in overweight, obesity and type 2 diabetes prevention: the research we should be doing | geo-analytics and intelligence for smarter public health decision-making[68] | big data  content: geo-analytics-based policies  overweight, obesity and type 2 diabetes prevention-based policies  lifestyle sensing-based policies  context: social (lifestyle) factor  process: agenda setting |
| 69 | Jean-Rémi Bourguet (2013) | An artificial intelligence-based approach to deal with argumentation applied to food quality in a public health policy | recommendation-based argumentation by extending Dung’s seminal argumentation system. new recommendations based on stakeholder’s argumentation by targeting some specific audiences[69] | Artificial Intelligence  Content: argumentation-based policies (obesity or diabetes)  Context: legal, political  Process: agenda setting |
| 70 | Changli Zhang1 (2008) | Polarity classification of public health opinions in Chinese | opinion mining in the context of public health opinions[70] | machine learning  Content: public health opinions -based policies  Context: legal, political  Process: agenda setting, implementation, evaluation |
| 71 | Erik NelsonI (2019) | The distributional impact of a green payment policy for organic fruit | impact of payment policies.  estimate the impact of the subsidy on organic fruit demand[71] | machine learning  Context: impact analysis of preventive interventions-based policies  Context: legal, political, economic  Process: agenda setting, evaluation |
| 72 | Bishwajit Nayak (2019  ) | [Democratizing health insurance services; accelerating social inclusion through technology policy of health insurance firms](https://www.scopus.com/record/display.uri?eid=2-s2.0-85068122544&origin=resultslist&sort=plf-f&src=s&sid=b851b7bbbbcf5e7aa4c1acaf85c08af4&sot=a&sdt=a&sessionSearchId=b851b7bbbbcf5e7aa4c1acaf85c08af4&relpos=67&citeCnt=7) | promote healthy lifestyles and disease prevention strategies to strengthen universal health care. determine optimum allocation of resources to areas that need priority[72] | big data  machine learning  content: social inclusion-based policies  context: social, technical  process: agenda setting |
| 73 | Paul van Schaik (2019  ) | [Explainable statistical learning in public health for policy development: The case of real-world suicide data](https://www.scopus.com/record/display.uri?eid=2-s2.0-85069474938&origin=resultslist&sort=plf-f&src=s&sid=b851b7bbbbcf5e7aa4c1acaf85c08af4&sot=a&sdt=a&sessionSearchId=b851b7bbbbcf5e7aa4c1acaf85c08af4&relpos=72&citeCnt=2) | improve the quality of health policy recommendations.  prediction identified four significant indicator predictors of suicide behaviour[73] | Machine learning  Content: behavior predict and online public health data analysis-based policies  Context: social  Process: agenda setting |
| 74 | Jung Wan LEE (2020) | Big Data Strategies for Government, Society and Policy-Making | help public administration, society, and policy-making to improve community’s lives.  more citizen-centric, responsive, accountable and transparent government services [74] | Content: citizen-centric, responsive, accountable and transparent policies  Context: legal |
| 75 | Maryam Zolnoori (2019) | Mining news media for understanding public health concerns | Mining news media for understanding public health concerns[75] | big data  Data mining  Content: public health concerns-based policies  Context: social factor  Process: agenda setting, implementation |
| 76 | FATIMA KHALIQUE (2021) | Multiple Disease Hotspot Mining for Public Health Informatics in Resource Starved Settings: Study of Communicable Diseases in Punjab, Pakistan | Disease Hotspot Mining. identification of locations with higher need of related public health resources[76] | Data mining  Content: optimal allocation of resources and Disease Hotspot Mining -based policies  Context: social  Process: agenda setting |
| 77 | Charles M. (2003) | Public health, GIS, and the Internet | disease surveillance, control, and prevention, and insure public access and community empowerment. use of GIS by public health and other sectors of the economy[77] | Data mining  Content: GIS based policies  Context: political, legal, social  Process: agenda setting, evaluation |
| 78 | Azra Ismail (2021) | AI in Global Health: The View from the Front Lines | Behavior Prediction,  Health Systems Measurement. Resource Allocation[78] | Artificial intelligence  Content: social good policies  Context: social, technical  process: agenda setting |
| 79 | Lester Darryl Geneviève (2022) | [Precision Public Health and Structural Racism in the United States: Promoting Health Equity in the COVID-19 Pandemic Response](https://www.scopus.com/record/display.uri?eid=2-s2.0-85125965063&origin=resultslist&sort=plf-f&src=s&nlo=&nlr=&nls=&sid=0512b12e36be5e7ef97b213d5fcab6ea&sot=a&sdt=a&sl=534&s=%28+%28+TITLE+%28+%22policy%22+%29+OR+TITLE+%28+%22global+health%22+%29+OR+TITLE+%28+%22public+health%22+%29+OR+TITLE+%28+%22international+health%22+%29+OR+TITLE+%28+%22policies%22+%29+OR+TITLE+%28+%22national+reform%22+%29+OR+TITLE+%28+%22priority+setting%22+%29+OR+TITLE+%28+%22national+program%22+%29+OR+TITLE+%28+%22public+administration%22+%29+OR+TITLE+%28+%22national+strategy%22+%29+%29+%29+AND+%28+TITLE+%28+health+%29+%29+AND+%28+%28+TITLE-ABS-KEY+%28+%22big+data%22+%29+OR+TITLE-ABS-KEY+%28+%22data+mining%22+%29+OR+TITLE-ABS-KEY+%28+%22artificial+intelligence%22+%29+OR+TITLE-ABS-KEY+%28+%22deep+learning%22+%29+OR+TITLE-ABS-KEY+%28+%22machine+learning%22+%29+%29+%29&relpos=13&citeCnt=0&searchTerm=) | structural inequalitie. Promoting Health Equity in the Pandemic Response[79] | machine learning  context: situational, social, political  content: social injustice and health inequity-based policies  process: agenda setting |
| 80 | Wan Rong (2021) | [Research on China's Tax Policy for Responding to Public Health Emergencies under the Background of Big Data](https://www.scopus.com/record/display.uri?eid=2-s2.0-85104542583&origin=resultslist&sort=plf-f&src=s&sid=41e6799fd5bcf297cdff81e2a617e7d9&sot=a&sdt=a&sessionSearchId=41e6799fd5bcf297cdff81e2a617e7d9&relpos=22&citeCnt=0) | New kinds of health financing (Tax Policy for Responding to Public Health Emergencies).  tax incentives that quickly responds to public health emergencies[80] | Big Data  Context: legal, Economic, Situational factors  Content: emergency Finance based policies  Process: agenda setting, implementation |
| 81 | Jerome amir singh (2019) | [Artificial Intelligence and global health: Opportunities and challenges](https://www.scopus.com/record/display.uri?eid=2-s2.0-85090905232&origin=resultslist&sort=plf-f&src=s&sid=9335cd0ddbcd43cdf7b8571a58c1feef&sot=a&sdt=a&sessionSearchId=9335cd0ddbcd43cdf7b8571a58c1feef&relpos=63&citeCnt=1) | contextually relevant knowledge, conclusions, and impactful actions. accelerate progress on achieving the United Nations' Sustainable Development Goals (SDGs) [81] | Content: SDG based policies  Context: international, legal, technical  Process: agenda setting |
| 82 | Roberto Aringhieri (2018) | [Evaluating the dispatching policies for a regional network of emergency departments exploiting health care big data](https://www.scopus.com/record/display.uri?eid=2-s2.0-85039413975&origin=resultslist&sort=plf-f&src=s&sid=de32465af2b2ceb898a62458c65b559d&sot=a&sdt=a&sessionSearchId=de32465af2b2ceb898a62458c65b559d&relpos=135&citeCnt=3) | health system analysis. Evaluating the dispatching policies[82] | Big Data  Content: dispatching evaluation-based policies  Context: political, legal  Process: evaluation |
| 83 | Khaiwal Ravindra (2016) | Air Pollution in India: Bridging the Gap between Science and Policy | actionable insights to facilitate policy decisions  predict the cost and benefits in terms of the control of acute and chronic diseases caused by air pollution. predict the cost of the air-pollution control measures[83] | Big Data  Content: science and policy gap-based policies  Context: technical, legal, political  Process: agenda setting, platform |
| 84 | Nadiya Straton (2016) | Big Social Data Analytics for Public Health: Facebook Engagement and Performance | Social Data Analytics. Engagement of social media users in public health[84] | machine learning  big data  content: social Engagement based policies  process: agenda setting  context: legal, political |
| 85 | Soojin Oh Park (2021) | What predicts legislative success of early care and education policies? Applications of machine learning and Natural Language Processing in a cross-state early childhood policy analysis | predicts legislative success[85] | Machine learning  Content: legislative effectiveness-based policies  Context: legal, political, social factors  Process: evaluation |
| 86 | Kevin Linka (2020) | The reproduction number of COVID-19 and its correlation with public health interventions | simulate various outbreak control and exit strategies[86] | Machine learning  Content: political based policies  Context: situational, international, political factor  Process: agenda setting |
| 87 | ZahidAlam Khan (2017) | Optimal Policy Learning for Disease Prevention Using Reinforcement Learning | Disease Prevention.Optimal Policy Learning[87] | deep learning  Big data  context: social, environment  content; Disease Prevention based policies  process: agenda setting (policy learning) |
| 88 | Jacqueline C.K (2019  0 | Stakeholder concerns of air pollution in Hong Kong and policy implications: A big-data computational text analysis approach | computational text analysis of Stakeholder to compare their concerns [88] | big data  context: political, environment, legal  content: Stakeholder concerns-based policies and interventions  process: agenda setting, implementation, evaluation |
| 89 | Leeann N (2020) | Do Longitudinal Trends in Tobacco 21-Related Media Coverage Correlate with Policy Support? an Exploratory Analysis Using Supervised and Unsupervised Machine Learning Methods | Policy Support analysis.  support impact for health policies by Media coverage[89] | Machine learning  process: Policy support for agenda setting  Content: regulation support-based policies  Context: social factor, political |
| 90 | Laura Mählmanna (2018) | Big Data for Public Health Policy-Making: Policy Empowerment | encourages citizens to participate and monitor policy changes and evaluate their impact and risk on a population level.  monitor policy changes and evaluate their impact and risk on a population level [90] | big data  context: legal, political factor  content: policy change monitor-based policies |

1. Jagrič, T., D. Fister, and V. Jagrič. Reshaping the Healthcare Sector with Economic Policy Measures Based on COVID-19 Epidemic Severity: A Global Study. in Healthcare. 2022. MDPI.

2. Wen, Z., et al., EpiTopics: A dynamic machine learning model to predict and inform non-pharmacological public health interventions from global news reports. STAR protocols, 2022. 3(2): p. 101463.

3. Bulut, O. and C.N. Poth, Rapid assessment of communication consistency: sentiment analysis of public health briefings during the COVID-19 pandemic. AIMS Public Health, 2022. 9(2): p. 293.

4. Martin-Moreno, J.M., et al., Predictive Models for Forecasting Public Health Scenarios: Practical Experiences Applied during the First Wave of the COVID-19 Pandemic. International Journal of Environmental Research and Public Health, 2022. 19(9): p. 5546.

5. Gauld, C., et al., Popular and Scientific Discourse on Autism: Representational Cross-Cultural Analysis of Epistemic Communities to Inform Policy and Practice. Journal of Medical Internet Research, 2022. 24(6).

6. Varela, A.R., et al., Prediction of SARS-CoV-2 infection with a Symptoms-Based model to aid public health decision making in Latin America and other low and middle income settings. Preventive medicine reports, 2022. 27: p. 101798.

7. Nath, B., et al., Predicting the distribution of arsenic in groundwater by machine learning technique in two worst hit districts of Assam, India: a risk to public health. medRxiv, 2022: p. 2021.12. 30.21268539.

8. Zhao, X. and G. Wang, Deep Q networks-based optimization of emergency resource scheduling for urban public health events. Neural Computing & Applications, 2022: p. 1-10.

9. Chen, K., C.S. Pun, and H.Y. Wong, Efficient Social Distancing during the COVID-19 Pandemic: Integrating Economic and Public Health Considerations. European Journal of Operational Research, 2021.

10. Zhong, H., et al., Metamodeling for Policy Simulations with Multivariate Outcomes. Medical Decision Making, 2022. 42(7): p. 872-884.

11. Oselio, B., et al., Reinforcement learning evaluation of treatment policies for patients with hepatitis C virus. BMC medical informatics and decision making, 2022. 22(1): p. 63-63.

12. Tian, H., Improvement of the Public Health Service Platform System Based on the Big Data-Driven System. 2022.

13. Farkhad, B.F., et al., State health policies and interest in PrEP: evidence from Google Trends. AIDS care, 2022. 34(3): p. 331-339.

14. Xu, Q., et al., Digital Surveillance to Identify California Alternative and Emerging Tobacco Industry Policy Influence and Mobilization on Facebook. International journal of environmental research and public health, 2021. 18(21): p. 11150.

15. Espinosa, L., et al., Epitweetr: Early warning of public health threats using Twitter data. Eurosurveillance, 2022. 27(39): p. 2200177.

16. Pathak, T.S., et al., Sentiments Evoked by WHO Public Health Posts During the COVID-19 Pandemic: A Neural Network-Based Machine Learning Analysis. 2021.

17. Zhang, Z., et al., The Impact of Public Health Events on COVID-19 Vaccine Hesitancy on Chinese Social Media: National Infoveillance Study. JMIR public health and surveillance, 2021. 7(11): p. e32936.

18. Buchbinder, M., et al., Advancing a Data Justice Framework for Public Health Surveillance. AJOB Empirical Bioethics, 2022: p. 1-9.

19. Prasinos, M., et al., A Modelling Framework for Evidence-Based Public Health Policy Making. IEEE Journal of Biomedical and Health Informatics, 2022. 26(5): p. 2388-2399.

20. Zhang, H. and T. Pan, Public Health Risk Assessment and Prevention Based on Big Data. Journal of Environmental and Public Health, 2022. 2022: p. 7965917-7965917.

21. Nijkamp, P. and K. Kourtit, Place-Specific Corona Dashboards for Health Policy: Design and Application of a ‘Dutchboard’. Sustainability, 2022. 14(2): p. 836.

22. Zhu, L., et al., Can artificial intelligence enable the government to respond more effectively to major public health emergencies?——Taking the prevention and control of Covid-19 in China as an example. Socio-Economic Planning Sciences, 2022. 80: p. 101029.

23. Basheer, M., et al., Balancing national economic policy outcomes for sustainable development. Nature communications, 2022. 13(1): p. 1-13.

24. Shi, X., et al., System Architecture of a European Platform for Health Policy Decision Making: MIDAS. Frontiers in public health, 2022. 10: p. 838438.

25. Evgeniou, T., et al., Pandemic lockdown, isolation, and exit policies based on machine learning predictions. Production and operations management, 2022.

26. Zhang, D., The Construction of National Image of China by English World Media in Public Health Emergencies. Journal of Environmental and Public Health, 2022. 2022.

27. Zhang, S., et al., Public View of Public Health Emergencies Based on Artificial Intelligence Data. Journal of Environmental and Public Health, 2022. 2022.

28. Wu, J.W., et al., Assessment of the Benefits of Targeted Interventions for Pandemic Control in China Based on Machine Learning Method and Web Service for COVID-19 Policy Simulation. Biomedical and Environmental Sciences, 2022. 35(5): p. 412-418.

29. Mbonyinshuti, F., et al., Application of random forest model to predict the demand of essential medicines for non-communicable diseases management in public health facilities. The Pan African Medical Journal, 2022. 42.

30. Ramírez, A.V., et al., Prediction of SARS-CoV-2 infection with a Symptoms-Based model to aid public health decision making in Latin America and other low and middle income settings. Preventive medicine reports, 2022. 27: p. 101798-101798.

31. Picco, G., et al., Learning Insurance Benefit Rules from Policy Texts with Small Labeled Data. Studies in health technology and informatics, 2022. 290: p. 292-296.

32. Wang, X., et al., From policy to prediction: Forecasting COVID-19 dynamics under imperfect vaccination. arXiv preprint arXiv:2201.05930, 2022.

33. Gao, Y., L. Zhu, and Z.J. Mao, How to improve public health literacy based on polycentric public goods theory: preferences of the Chinese general population. BMC public health, 2022. 22(1): p. 1-13.

34. Basheer, M., et al., Balancing national economic policy outcomes for sustainable development. Nature Communications, 2022. 13: p. 5041.

35. Lucero‑Obusan, C., et al., Public health surveillance in the US Department of Veterans Affairs: evaluation of the Praedico surveillance system. 2022.

36. Wen, Z., et al., Inferring global-scale temporal latent topics from news reports to predict public health interventions for COVID-19. Patterns, 2022. 3(3): p. 100435.

37. Edwards, D.J., Ensuring effective public health communication: Insights and modeling efforts from theories of behavioral economics, heuristics, and behavioral analysis for decision making under risk. Frontiers in Psychology, 2021. 12.

38. Kniesner, T.J. and J.D. Leeth, Data mining mining data: MSHA enforcement efforts, underground coal mine safety, and new health policy implications. Journal of Risk and Uncertainty, 2004. 29(2): p. 83-111.

39. Robb, K., et al., Using integrated city data and machine learning to identify and intervene early on housing-related public health problems. Journal of Public Health Management and Practice, 2022. 28(2): p. E497.

40. Moutselos, K. and I. Maglogiannis, Evidence-based Public Health Policy Models Development and Evaluation using Big Data Analytics and Web Technologies. Medical Archives, 2020. 74(1): p. 47.

41. Mavrogiorgou, A., et al. CrowdHEALTH: An e-Health Big Data Driven Platform towards Public Health Policies. in ICT4AWE. 2020.

42. Kyriazis, D., et al., The CrowdHEALTH project and the hollistic health records: Collective wisdom driving public health policies. Acta Informatica Medica, 2019. 27(5): p. 369.

43. Vassiliou, A.G., et al., Health in all policy making utilizing big data. Acta Informatica Medica, 2020. 28(1): p. 65.

44. KBioAssist, S., et al., Crowdhealth: Holistic health records and big data analytics for health policy making and personalized health. Informatics Empowers Healthcare Transformation, 2017. 238: p. 19.

45. Cleland, B., et al. Usability evaluation of a co-created big data analytics platform for health policy-making. in International Conference on Human-Computer Interaction. 2019. Springer.

46. Zhou, J., et al., Framework construction and application for global health information platform. Wuhan University Journal of Natural Sciences, 2015. 20(2): p. 153-158.

47. Moutselos, K., D. Kyriazis, and I. Maglogiannis. A web based modular environment for assisting health policy making utilizing big data analytics. in 2018 9th International Conference on Information, Intelligence, Systems and Applications (IISA). 2018. IEEE.

48. Spanoudakis, G., et al. Public health policy for management of hearing impairments based on big data analytics: EVOTION at Genesis. in 2017 IEEE 17th International Conference on Bioinformatics and Bioengineering (BIBE). 2017. IEEE.

49. Buchan, I., et al., IMPACT: A generalisable system for simulating public health interventions, in MEDINFO 2010. 2010, IOS Press. p. 486-490.

50. Anisetti, M., et al. Big data platform for public health policies. in 2017 IEEE SmartWorld, Ubiquitous Intelligence & Computing, Advanced & Trusted Computed, Scalable Computing & Communications, Cloud & Big Data Computing, Internet Of People And Smart City Innovation (SmartWorld/SCALCOM/UIC/ATC/CBDCom/IOP/SCI). 2017. IEEE.

51. Ainsworth, J., et al., IMPACT: a generic tool for modelling and simulating public health policy. Methods of information in medicine, 2011. 50(05): p. 454-463.

52. Moore, M., et al., Strategies to improve global influenza surveillance: a decision tool for policymakers. BMC Public Health, 2008. 8(1): p. 1-11.

53. Li, Y. and L. Xiao, Research on public health crisis early warning system based on context awareness. Technology and Health Care, 2022(Preprint): p. 1-12.

54. Saunders, G.H., et al., Application of big data to support evidence-based public health policy decision-making for hearing. Ear and hearing, 2020. 41(5): p. 1057.

55. Zolbanin, H.M., D. Delen, and S.K. Sharma, The strategic value of big data analytics in health care policy-making. International Journal of E-Business Research (IJEBR), 2018. 14(3): p. 20-33.

56. Lu, X.H., et al. Guiding public health policy by using grocery transaction data to predict demand for unhealthy beverages. in International Workshop on Health Intelligence. 2019. Springer.

57. English, N., et al., Image Processing for Public Health Surveillance of Tobacco Point-of-Sale Advertising: Machine Learning–Based Methodology. Journal of Medical Internet Research, 2021. 23(8): p. e24408.

58. Yang, W., et al., A novel framework for forecasting, evaluation and early-warning for the influence of PM10 on public health. Atmosphere, 2021. 12(8): p. 1020.

59. Jo, H.S. and S.M. Jung, Evaluation of food labeling policy in Korea: analyzing the Community Health Survey 2014–2017. Journal of Korean Medical Science, 2019. 34(32).

60. Hamad, R., et al., Large‐scale automated analysis of news media: a novel computational method for obesity policy research. Obesity, 2015. 23(2): p. 296-300.

61. Kreif, N., et al., Estimating heterogeneous policy impacts using causal machine learning: a case study of health insurance reform in Indonesia. Health Services and Outcomes Research Methodology, 2022. 22(2): p. 192-227.

62. Ji, X., S.A. Chun, and J. Geller. Monitoring public health concerns using twitter sentiment classifications. in 2013 IEEE International Conference on Healthcare Informatics. 2013. IEEE.

63. Barfar, A. and B. Padmanabhan, Pattern discovery, validation, and online experiments: a methodology for discovering television shows for public health announcements. Journal of the American Medical Informatics Association, 2021. 28(7): p. 1374-1382.

64. Xie, W., et al., Developing Machine Learning and Statistical Tools to Evaluate the Accessibility of Public Health Advice on Infectious Diseases among Vulnerable People. Computational Intelligence and Neuroscience, 2021. 2021.

65. Lamprell, G. and J. Braithwaite, Mainstreaming gender and promoting intersectionality in Papua New Guinea’s health policy: a triangulated analysis applying data-mining and content analytic techniques. International Journal for Equity in Health, 2017. 16(1): p. 1-10.

66. Chae, Y.M., et al., Data mining approach to policy analysis in a health insurance domain. International journal of medical informatics, 2001. 62(2-3): p. 103-111.

67. Wali, B., et al., Developing policy thresholds for objectively measured environmental features to support active travel. Transportation research part D: transport and environment, 2021. 90: p. 102678.

68. Kamel Boulos, M.N. and K. Koh, Smart city lifestyle sensing, big data, geo-analytics and intelligence for smarter public health decision-making in overweight, obesity and type 2 diabetes prevention: the research we should be doing. 2021, Springer. p. 1-10.

69. Bourguet, J.-R., et al., An artificial intelligence-based approach to deal with argumentation applied to food quality in a public health policy. Expert Systems with Applications, 2013. 40(11): p. 4539-4546.

70. Zhang, C., et al. Polarity classification of public health opinions in Chinese. in International Conference on Intelligence and Security Informatics. 2008. Springer.

71. Nelson, E., J. Fitzgerald, and N. Tefft, The distributional impact of a green payment policy for organic fruit. PloS one, 2019. 14(2): p. e0211199.

72. Nayak, B., S.S. Bhattacharyya, and B. Krishnamoorthy, Democratizing health insurance services; accelerating social inclusion through technology policy of health insurance firms. Business Strategy & Development, 2019. 2(3): p. 242-252.

73. Van Schaik, P., et al., Explainable statistical learning in public health for policy development: the case of real-world suicide data. BMC medical research methodology, 2019. 19(1): p. 1-14.

74. Lee, J.W., Big data strategies for government, society and policy-making. Lee, Jung Wan (2020). Big Data Strategies for Government, Society and Policy-Making. Journal of Asian Finance Economics and Business, 2020. 7(7): p. 475-487.

75. Zolnoori, M., et al., Mining news media for understanding public health concerns. Journal of clinical and translational science, 2021. 5(1).

76. Khalique, F. and S.A. Khan, Multiple Disease Hotspot Mining for Public Health Informatics in Resource Starved Settings: Study of Communicable Diseases in Punjab, Pakistan. IEEE Access, 2021. 9: p. 89989-89998.

77. Croner, C.M., Public health, GIS, and the Internet. Annual Review of Public Health, 2003. 24: p. 57.

78. Ismail, A. and N. Kumar. AI in global health: the view from the front lines. in Proceedings of the 2021 CHI Conference on Human Factors in Computing Systems. 2021.

79. Geneviève, L.D., et al., Precision Public Health and Structural Racism in the United States: Promoting Health Equity in the COVID-19 Pandemic Response. JMIR Public Health and Surveillance, 2022. 8(3): p. e33277.

80. Wang, W.R. Research on China's Tax Policy for Responding to Public Health Emergencies under the Background of Big Data. in 2020 International Conference on Modern Education and Information Management (ICMEIM). 2020. IEEE.

81. Singh, J.A., Artificial intelligence and global health: opportunities and challenges. Emerging Topics in Life Sciences, 2019. 3(6): p. 741-746.

82. Aringhieri, R., et al. Evaluating the dispatching policies for a regional network of emergency departments exploiting health care big data. in International Workshop on Machine Learning, Optimization, and Big Data. 2017. Springer.

83. Ravindra, K., et al., Air pollution in India: bridging the gap between science and policy. Journal of Hazardous, Toxic, and Radioactive Waste, 2016. 20(4): p. A4015003.

84. Straton, N., et al. Big social data analytics for public health: Facebook engagement and performance. in 2016 IEEE 18th International Conference on e-Health Networking, Applications and Services (Healthcom). 2016. IEEE.

85. Park, S.O. and N. Hassairi, What predicts legislative success of early care and education policies?: Applications of machine learning and Natural Language Processing in a cross-state early childhood policy analysis. Plos one, 2021. 16(2): p. e0246730.

86. Linka, K., M. Peirlinck, and E. Kuhl, The reproduction number of COVID-19 and its correlation with public health interventions. Computational Mechanics, 2020. 66(4): p. 1035-1050.

87. Alam Khan, Z., et al., Optimal policy learning for disease prevention using reinforcement learning. Scientific Programming, 2020. 2020.

88. Lam, J.C., et al., Stakeholder concerns of air pollution in Hong Kong and policy implications: A big-data computational text analysis approach. Environmental Science & Policy, 2019. 101: p. 374-382.

89. Siegel, L.N., et al., Do longitudinal trends in tobacco 21-Related media coverage correlate with policy support? an exploratory analysis using supervised and unsupervised machine learning methods. Health Communication, 2022. 37(1): p. 29-38.

90. Mählmann, L., et al., Big data for public health policy-making: policy empowerment. Public health genomics, 2017. 20(6): p. 312-320.
